# Measuring Self-Directed Readiness (SDLR) of internal medicine trainees and the impact of covid pandemic on their training and learning acquisitions cross-sectional study

**DOI:** 10.1097/MS9.0000000000003722

**Published:** 2025-09-02

**Authors:** Jawahir M. Abuhaimed, Yazid Alhadlg, Abdullah Altwaim, Hatem Alnasser

**Affiliations:** a College of Medicine, AL Faisal University, Riyadh, Saudi Arabia; b Internal Medicine Department, Nephrology Unit, King Fahad Medical City, Riyadh, Saudi Arabia; c College of Medicine, King Saud University, Riyadh, Saudi Arabia; d Internal Medicine Department, Nephrology Unit, King Saud University Medical City, Riyadh, Saudi Arabia.

**Keywords:** COVID-19, internal medicine trainee, self-directed learning readiness

## Abstract

**Introduction:**

The COVID-19 pandemic has profoundly affected medical education worldwide, leading to significant disruptions in healthcare systems. This study investigates the impact of the pandemic on the readiness for self-directed learning (SDL) among first-year, third-year, and fourth-year internal medicine residents in Saudi Arabia during the 2019–2020 academic year.

**Methods:**

A non-interventional cross-sectional study utilizing an online survey based on SDL concepts was conducted in Saudi Arabia in 2022-2023. Data were collected from 74 participants with ethical approval, and analysis involved statistical testing and questionnaire reliability evaluation.

**Results:**

Overall, participants exhibited high SDL readiness scores, indicating readiness for self-directed learning. Gender, Part 1 residency exam results, and effective knowledge sources did not significantly affect SDL preparedness. However, residents who dedicated eight or more hours weekly to reading demonstrated higher SDL readiness, emphasizing a strong commitment to self-directed learning. Choosing textbooks as a primary knowledge source was associated with increased SDL readiness scores. Although first-year residents showed higher SDL readiness ratings than senior fellows, this difference was not statistically significant. Notably, residents exhibited increased engagement in reading patient encounters.

**Conclusions:**

Despite pandemic challenges, Saudi Arabian internal medicine residents maintained high SDL readiness. The pandemic did not substantially alter SDL preparation concerning information sources, gender, or exam scores. Reading habits indicative of self-directed learning commitment positively influenced SDL preparedness. Larger sample sizes may be necessary to definitively assess SDL preparedness differences between first-year residents and the third- to fourth-year colleagues. These findings underscore the adaptability and resilience of medical students during global health crises, highlighting the importance of self-directed learning in medical education.

## Introduction

On 11 March 2020, the World Health Organization (WHO) declared the COVID-19 outbreak a global pandemic. By 27 May 2020, Saudi Arabia had reported over 76,000 cases and 41 deaths. In response, healthcare institutions had to quickly reorganize their infrastructure to effectively handle the crisis, optimize resources, and prevent the further spread of the virus^[1]^. As a result of the pandemic, junior doctors’ training has been significantly impacted, with more than 80% of residents reporting interruptions in their training due to the pandemic, according to the 2020 General Medical Council (GMC) national training survey^[2]^. Concerns about the well-being of these trainees have been raised due to the disruption in training and the increased demand for patient care^[3,4]^. The working environment for healthcare personnel in the UK has also undergone significant changes, including the implementation of social distancing guidelines and the use of personal protective equipment. Some positive outcomes amidst the crisis include the adoption of new virtual training techniques and the transition to online outpatient clinics^[5]^. Instead of large group didactic sessions, bedside just-in-time learning became more popular, with faculty members and residents using instant messaging services like WhatsApp to discuss COVID-19-related topics. Additionally, training programs quickly implemented measures to minimize risk for residents, such as providing refresher courses on the proper usage of personal protective equipment, updates on hospital operations, and protocols for handling suspected and confirmed cases^[6]^. Unfortunately, the pandemic has led. to a reduction in valuable educational opportunities, with fewer clinical cases and elective services being discontinued or switched to telephone or virtual formats^[7]^. Despite these challenges, the ability to find innovative and adaptable solutions during times of crisis is evidence of a capacity to bring about positive change^[2]^. Many non-specialized doctors and surgeons have had to dedicate a significant portion of their time to treating COVID-19 patients, resulting in a significant reduction in clinical and surgical practice across all Saudi Arabian institutions. This situation has also had a severe impact on the training of residents and fellows^[1]^. To ensure effective and efficient training, self-directed learning has been emphasized for medical students, residents, practicing physicians, nurses, and other healthcare workers. Self-directed learning is seen as crucial for maintaining professionalism in medicine and requires possessing the attitudes, skills, and personality traits required for independent learning^[7]^. In higher education, students are increasingly expected to take greater responsibility for their own learning by exploring subjects that align with their curiosity, interests, and abilities. This approach helps nurture their full potential and talents. In this context, educators play a pivotal role as facilitators and mentors, guiding students toward deeper and more meaningful learning experiences. Self-directed learning (SDL) supports both personal and professional development by equipping students with essential knowledge and skills^[8]^. The Self-Directed Learning Readiness (SDLR) Scale is a validated instrument used to assess an individual’s readiness for self-directed learning. Beyond traditional methods like reading textbooks and participating in workshops or seminars, advancements in information technology have enabled students to cultivate self-directed learning skills through their preferred formats or platforms—such as exploring relevant websites, accessing e-books, and viewing educational content on YouTube^[9]^. Two versions of the SDLR scale, created by Guglielmino in 1977 and Fisher in 2001, have been used to investigateself-directed learning in various fields, including non-medical education, nursing, pharmacy,and medicine^[10]^. Fisher’s scale consists of three components: self-management, learningmotivation, and self-control. A score above 150 on Fisher’s 40-item scale, which has a maximum score of 200, indicates a high level of preparedness for self-directed learning^[11,12]^. This scale has been validated by medical students^[10]^ The ability to engage in self-directed learning is crucial for strengthening competence in both knowledge and skills. To support this, a variety of teaching approaches have been implemented to help students develop SDL capabilities, preparing them to navigate the fast-paced and ever-evolving healthcare environment^[8]^This study examined the Self-Directed Learning Readiness (SDLR) scores of first-year as well as third- and fourth-year internal medicine residents during the 2019–2020 academic year to explore the impact of the COVID-19 pandemic on their training experiences and learning acquisition. Specifically, it aimed to assess whether the pandemic influenced SDL characteristics and behaviors among internal medicine trainees in Saudi Arabia, and whether the resulting disruptions to their clinical education affected their readiness for self-directed learning.

**HIGHLIGHTS**

- Despite disruptions caused by the pandemic, residents demonstrated high SDL readiness, especially those who spent more time reading or preferred textbooks as learning sources.
- No significant differences were observed in SDL readiness based on gender, exam performance, or primary learning source, suggesting pandemic resilience in educational engagement.

- The results emphasize the importance of institutional support, digital learning platforms, and individual motivation in maintaining SDL during global health crises.

## Methods

### Study design

This study adopted a non-interventional, cross-sectional design, conducted to explore the impact of the COVID-19 pandemic on the self-directed learning readiness (SDLR) of internal medicine trainees. The setting of the study included educational centers across Riyadh, Saudi Arabia, during the academic years 2022–2023. An online questionnaire, based on the concepts of self-directed learning proposed by Garrison, was utilized. Fisher’s validated Self-Directed Learning Readiness (SDLR) scale was used, identifying three core components: self-management, learning motivation, and self-control^[13,14]^. The instrument was structured on a Likert scale and delivered via Google Forms.

The SDLR tool used in this study was the validated 40-item Fisher’s Self-Directed Learning Readiness Scale, which has been applied in various educational contexts. The tool was used without cultural adaptation or translation, as the participants were fluent in English and the training program operates in English. However, cultural differences may influence interpretation of some items.

The questionnaire was disseminated through mass text messages (using a texting app) and email invitations, which were sent to all eligible trainees. The text included a link to the online form, which first presented an informed consent section, followed by the survey itself. The **target population** included first-year internal medicine trainees from the 2019–2020 academic year and their senior counterparts, defined as those in their third and fourth years during the same time period. **Medical interns and trainees who had withdrawn** from the program were excluded from the study.

Upon concluding data collection, responses were securely stored on a private **Cloud server** with **limited access**, restricted to the principal investigator, ensuring confidentiality and data protection. This work has been reported in line with the **STROCSS 2025 guidelines**^[15]^, ensuring transparency and adherence to scientific reporting standards.

## Ethical approval

Ethical approval was granted by the ethical standards of the Research Ethics Committee under the **Institutional Review Board (IRB)** number **(E−22-7238)**, issued on **late 2022**. All participants provided informed consent prior to their participation and were informed of their right to withdraw from the study at any point before the data analysis stage.

### Statistical analysis

Data were analyzed by using Statistical Package for Social Studies (SPSS 22; IBM Corp., New York, NY, USA). Continuous variables were expressed as mean ± standard deviation and categorical variables were expressed as percentages. Both the Mann-Whitney Test and the Kruskal-Wallis Test were used for continuous variables. Cronbach’s alpha was used to assess the reliability of the questionnaire. A p-value <0.05 was considered statistically significant.

### Reliability of the questionnaire

The internal consistency of the instrument was measured using **Cronbach’s alpha**, yielding excellent reliability across all items. The Cronbach’s alpha scores for the overall scale and each subscale were as follows: overall questionnaire—**0.935**, self-management—**0.835**, self-control—**0.895**, and desire for learning—**0.884**. These values indicate high internal consistency and reliability, as presented in Supplementary digital content Table 1, available at: http://links.lww.com/MS9/A927).

## Results

In the study, Supplementary digital content Table 2, available at: http://links.lww.com/MS9/A927 illustrates that a total of 74 participants were involved. Females and males comprised 52.7% (n = 39) and 47.3% (n = 35) of the participants, respectively. Among them, 39.19% (n = 29) were internal medicine residents who enrolled in 2019–2020, which was the academic year when the pandemic began. In the academic year 2019–2020, 89.66% (n = 66) of the participants took their Part 1 exam, while 10.34% (n = 8) attempted it in 2020–2021. Among the senior fellows who took their Part 1 exam in the year 2016–2017, the majority (56.41%, n = 22) were successful. When it came to the study habits of the participants, 85.14% (n = 63) passed their Part 1 exam on the first attempt, while 14.86% (n = 11) did not. A notable portion of the trainees dedicated different amounts of time to reading during their practice, with 29.73% (n = 22) spending 3–4 hours, 18.92% (n = 14) spending 1–2 hours, 16.22% (n = 12) spending 5–6 hours, and 17.57% (n = 13) spending over 8 hours weekly on reading.

Overall, the trainees strongly agreed (43.24%, n = 32) that their readings were primarily triggered by patient encounters, while 24.32% (n = 18) agreed and 25.68% (n = 19) remained neutral on the matter. The most effective sources of knowledge for the trainees were UpToDate (55.41%, n = 41), textbooks (24.32%, n = 18), and patient care/bedside rounds (9.46%, n = 7).

Supplementary digital content Table 3, available at: http://links.lww.com/MS9/A927 displays the scores for each item related to readiness for self-directed learning (SDL). In the self-control domain, the highest scores were obtained for the 3rd, 11th, 13th, and 14th items, with scores of 4.36 ± 1.09, 4.27 ± 0.80, 4.23 ± 0.96, and 4.23 ± 0.93, respectively. For the self-management domain, the highest scores were achieved for the 8th, 1st, and 13th items, with scores of 4.05 ± 0.92, 4.03 ± 0.90, and 3.95 ± 0.89, respectively. Lastly, in the desire for learning domain, the highest scores were obtained for the 4th, 3rd, 12th, and 10th items, with scores of 4.46 ± 0.79, 4.31 ± 1.00, 4.31 ± 0.89, and 4.31 ± 0.77, respectively.

The overall SDL readiness scores, as shown in Supplementary digital content Table 4, available at: http://links.lww.com/MS9/A927, were 160.85 ± 17.58 out of 200. In the self-control domain, the score was 63.13 ± 7.85 out of 75. The self-management scale yielded a score of 47.73 ± 6.41 out of 65. Lastly, the desire for learning domain received a score of 49.99 ± 5.22 out of 60.

The participants’ characteristics were analyzed in Supplementary digital content Table 5, available at: http://links.lww.com/MS9/A927 to determine their SDL readiness score using the following variables: gender, Part 1 residency exam results, effective source of knowledge, and time spent reading each week. The results showed that there was no significant difference in SDL readiness score based on gender (p = 0.391), Part 1 residency exam results (p = 0.473), and the effective source of knowledge (p = 0.230). However, the analysis did reveal a significant result for the category of time (hours) spent on reading each week, with a p-value of 0.023. When examining the gender variable, it was found that 77.14% (n = 27) of male residents had scores greater than 150, while females did not show a significant difference in scoring above 150. Interestingly, trainees who spent 8 or more hours on reading each week achieved 100% (n = 13) scores above 150. Furthermore, when trainees were asked about the most effective source of knowledge, 94.44% (n = 17) of those who chose textbooks as their preferred source had scores above 150.

Out of all internal medicine residents, 82.76% (n = 48) had scores greater than 150 compared to their senior fellows, who had a 73.33% (n = 22) self-directed learning (SDL) score. However, this difference was not found to be statistically significant, as indicated by a p-value of 0.215, which is represented in Supplementary digital content Table 6, available at: http://links.lww.com/MS9/A927.

The provided subscale in Supplementary digital content Figure 1, available at: http://links.lww.com/MS9/A927 represents the mean scores for different domains related to SDL readiness (SDLR), categorized by gender. The figure breaks down the mean scores across three specific domains: self-control, self-management, and desire for learning, comparing the scores between males and females. The data suggests that, on average, males expressed slightly higher scores in the three domains in the context of self-directed learning readiness compared to females. These findings provide insights into potential gender differences in SDLR, though no significant statistical difference was observed.

Supplementary digital content Table 7, available at: http://links.lww.com/MS9/A927 presents a comparison of learning habits between residents and senior fellows, showing a statistically significant result in their patient encounter-driven reading, with a p-value of 0.020.

## Discussion

The COVID-19 pandemic has raised unprecedented challenges for global healthcare systems. This study aimed to investigate the impact of the pandemic on the readiness for self-directed learning (SDL) among first-year and senior third and fourth-year internal medicine residents in the academic year 2019-2020. The results shed light on the SDL readiness of these trainees and provide insights into how the pandemic affected their training and learning acquisition. The findings of this study revealed that the overall SDL readiness scores for the participants were generally high. This suggests that, despite the disruptions caused by the pandemic, junior internal medicine residents demonstrated a strong preparedness for self-directed learning. This contradicts previous findings in which trainees in Saudi Arabia experienced varying degrees of impact, often leading to emotional disturbance, which is recognized as a common consequence of catastrophic events, health challenges, shifts in behavior, and emotional distress^[1,13,14]^. The results also indicated that there were no significant differences in SDL readiness scores based on gender, which is consistent with several studies^[7,16,17]^, However, some studies have reported differing results, indicating that females showed significant improvement in SDL—particularly in self-control—along with higher cognitive awareness, stronger motivation, better time management, and a greater tendency for self-initiated learning^[18]^, Part 1 residency exam results, and the effective source of knowledge categories. This suggests that the pandemic did not significantly impact these factors when it comes to SDL readiness. However, the analysis did reveal a significant result in the category of time spent reading each week, with trainees who spent 8 hours or more on reading having higher SDL readiness scores despite the disruption of the pandemic. A previous study mentioned that the trainees rarely had enough time to read and study for the Saudi Council for Health Specialties (SCFHS) exam during the pandemic^[1]^. A higher commitment to self-directed learning, including extensive reading, might enhance SDL readiness, irrespective of the pandemic’s influence. The choice of textbooks as the most effective source of knowledge was associated with higher SDL readiness scores. This finding underscores the importance of traditional educational resources in the SDL process.

One noteworthy finding was that, in comparison to their senior fellows, a higher percentage of first-year internal medicine residents had SDL readiness ratings above 150. However, this difference was not statistically significant. While data suggests that neither group’s level of SDL preparedness was greatly impacted by the pandemic, it is still important to investigate the causes of the variations in SDL readiness between first-year residents and senior fellows. This might be due to the small sample size, further analysis, or a larger sample size may be needed to draw a more definitive conclusion.

Additionally, when examining the learning behaviors of residents and senior fellows, a notable statistically significant disparity emerged in their engagement with patient encounter reading, as indicated by a p-value of 0.020. This conclusion is consistent with earlier studies that have developed the idea of integrated learning programs. Learners tend to be more inclined toward self-directed learning when they are exposed to new clinical cases or unusual situations^[7,19]^.

The findings of this study highlight the resilience and adaptability of internal medicine residents in the face of a global health crisis. It is encouraging to see that despite the challenges posed by the COVID-19 pandemic, these trainees maintained a strong readiness for self-directed learning. the SDLR tool used in this study was applied without cultural or linguistic modification, as the language of instruction for the training program was English and all participants were proficient in it. While internal consistency was strong in our results, the lack of cultural adaptation may still influence how some items are interpreted in the Saudi Arabian context. Future research should consider localized validation of the SDLR tool to ensure its applicability and sensitivity across diverse educational and cultural environments. However, it is important to continue investigating a larger sample size and implement longitudinal studies that could provide deeper insights into the trajectory of self-directed learning readiness amidst prolonged crises.

## Conclusion

This study offers a valuable exploration of self-directed learning readiness among internal medicine residents in Saudi Arabia during the COVID-19 pandemic. The findings suggest that SDL remains robust in times of crisis, particularly when supported by access to digital resources and adaptive learning strategies. To enhance the generalizability of these results, future research should focus on larger and multi-institutional samples, while incorporating longitudinal designs to capture changes in SDL over time. Strengthening SDL frameworks in residency training could improve resilience in future healthcare disruptions.

## Data Availability

The datasets generated and analyzed during the current study are not publicly available for Confidentiality reasons but are available from the corresponding author upon reasonable request.

## Research registration unique identifying number (UIN)

Not required.
